# All-Natural Gelatin-Based Nanoemulsion Loaded with TLR 7/8 Agonist for Efficient Modulation of Macrophage Polarization for Immunotherapy

**DOI:** 10.3390/nano14191556

**Published:** 2024-09-26

**Authors:** Ritabrita Goswami, Ahmed Nabawy, Mingdi Jiang, Yagiz Anil Cicek, Muhammad Aamir Hassan, Harini Nagaraj, Xianzhi Zhang, Vincent M Rotello

**Affiliations:** Department of Chemistry, University of Massachusetts Amherst, 710 North Pleasant Street, Amherst, MA 01003, USAmingdijiang@umass.edu (M.J.); ycicek@umass.edu (Y.A.C.); hnagaraj@umass.edu (H.N.);

**Keywords:** macrophage, immunotherapy, biopolymers, nanoemulsion, cancer immunotherapy

## Abstract

Macrophages are multifunctional immune cells essential for both innate and adaptive immune responses. Tumor-associated macrophages (TAMs) often adopt a tumor-promoting M2-like phenotype, aiding tumor progression and immune evasion. Reprogramming TAMs to a tumoricidal M1-like phenotype is an emerging target for cancer immunotherapy. Resiquimod, a TLR7/8 agonist, can repolarize macrophages from the M2- to M1-like phenotype but is limited by poor solubility. We developed a gelatin nanoemulsion for the loading and delivery of resiquimod, utilizing eugenol oil as the liquid phase and riboflavin-crosslinked gelatin as a scaffold. These nanoemulsions showed high stability, low toxicity, and effective macrophage repolarization, significantly enhancing pro-inflammatory markers and anticancer activity in co-culture models.

## 1. Introduction

Macrophages are highly plastic immune cells that play crucial roles in innate and adaptive immunity against tumors and infectious diseases [1,2,3]. These cells can differentiate into a broad array of phenotypes that can be categorized into two broad families: M1-like (classically activated) and M2-like (alternatively activated) macrophages [4,5,6,7]. M1-like macrophages, usually activated by interferon-gamma (IFN-γ) and lipopolysaccharide (LPS), are typically involved in pro-inflammatory responses, producing reactive oxygen and nitrogen species and phagocytosing antigens to combat pathogens and tumors [8,9]. Conversely, M2-like macrophages, typically activated by interleukins like interleukin-4 (IL-4) and interleukin-13 (IL-13), mediate anti-inflammatory processes, promoting wound healing and promoting angiogenesis [10,11,12].

Many cancers can manipulate macrophages within the tumor environment, converting them into tumor-associated macrophages (TAMs) [13,14,15]. Tumor-associated macrophages (TAMs) primarily exhibit a tumor-promoting M2-like phenotype that significantly contributes to tumor progression, metastasis, immune system evasion, and resistance to immunotherapy [16,17]. These TAMs can constitute a major part of the tumor mass (~50%) [18,19]. Significantly, TAMs can interfere with chemotherapy by absorbing the drugs meant for cancer cells [20,21,22].

Reprogramming of TAMs from M2-like pro-tumoral phenotypes to tumoricidal M1-phenotypes has emerged as a promising strategy in cancer immunotherapy [23]. Toll-like receptors (TLRs) are among the most utilized targets for immunomodulation owing to their high expression in immune cells and various tumor cells [24,25]. Toll-like receptor 7 and 8 (TLR7/8) targeting with concomitant TAM reprogramming has shown promise in various preclinical and early clinical studies [26,27]. Resiquimod, a potent TLR 7 and 8 (TLR7/8) agonist, is an FDA-approved immunomodulator that can polarize macrophages into an M1-like phenotype [28]. However, poor aqueous solubility limits the clinical use of resiquimod to topical applications only [29].

Synthetic nanomaterials, including polymers and nanoparticles, provide robust platforms for encapsulating and delivering resiquimod [30,31,32,33]. However, the utilization of non-biodegradable delivery scaffolds in clinical settings faces the challenges of non-specific tissue accumulation and potentially serious adverse effects [34,35]. Biopolymers provide efficient nanocarriers for the delivery of poorly soluble drugs, offering the advantages of biodegradability and limited immunogenicity [36,37,38,39,40].

**Figure 1:**
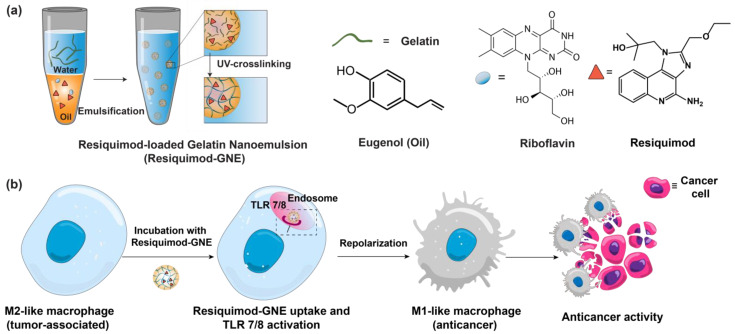
(**a**) Fabrication of Resiquimod-GNE nanoemulsion. The components are gelatin, eugenol, riboflavin, and resiquimod. (**b**) Schematic of macrophage reprogramming through TLR7/8 activation facilitated by Resiquimod-GNE.

Here, we report an all-natural biopolymer-based nanoemulsion platform loaded with TLR7/8 agonist resiquimod for efficient repolarization of tumor-associated macrophages into the M1 pro-inflammatory phenotype (Figure 1). In this delivery platform, crosslinked gelatin is used as an amphiphilic biopolymer scaffold to formulate a nanoemulsion with eugenol, a naturally derived essential oil. Loading the TLR7/8 agonist resiquimod into the oil core of the gelatin nanoemulsion provides a modular and biodegradable immunomodulatory nanotherapeutic that is promising for clinical translation. Resiquimod-loaded gelatin nanoemulsions (Resiquimod-GNE) demonstrated high stability and minimal mammalian cell toxicity but were rapidly degraded with collagenase enzymes. Resiquimod-GNE rapidly penetrated and accumulated inside M2-like macrophages, leading to efficient repolarization into M1-like phenotypes, significantly more than resiquimod alone, as evidenced by the upregulation of pro-inflammatory markers. The anticancer potential of Resiquimod-GNE was demonstrated by its ability to induce repolarization of M2-like macrophages, which resulted in effective cancer cell destruction in a macrophage-cancer cell co-culture model.

## 2. Materials and Methods

### 2.1. Materials

All materials were purchased from Fisher Scientific (Hampton, NH, USA) or Sigma-Aldrich (St. Louis, MO, USA) and used without additional purification unless otherwise noted. RAW 264.7 cells and GFP-U2OS cells were sourced from American Type Culture Collection (ATCC) (Manassas, VA, USA) and maintained in high-glucose Dulbecco’s Modified Eagle Medium (DMEM) and 10% fetal bovine serum (FBS), and 1% antibiotics were added.

### 2.2. Preparation of Resiquimod-Loaded Gelatin Nanoemulsions

Resiquimod-GNEs were fabricated through emulsification of eugenol suspension loaded with resiquimod and riboflavin (vitamin B2) with an aqueous solution of gelatin. Initially, riboflavin (1 mg/mL) and resiquimod (5 mg/mL) were dissolved in eugenol. Then, 3 μL of this resulting oil mixture was added to 497 μL of an aqueous gelatin solution (240 mg/L). The mixture was emulsified for 1 min using a Zoneray HL-AH G8 amalgamator (Treedental, Rockland, NY, USA). Upon emulsification, gelatin self-assembled at the interface between eugenol oil and water. UV irradiation at 365 nm using a 3UV Lamp (115V, 60Hz, Cat #95034) (Fisher Scientific, Hampton, NH, USA) was then used for 25 min to crosslink the gelatin, producing a stable nanoemulsion.

### 2.3. Size and Charge of Nanoemulsions by Dynamic Light Scattering (DLS)

A Zetasizer Nano ZS instrument (Malvern Panalytical, Malvern Borough, PA, USA) at a 173° backscatter angle was used to evaluate the size and charge of the nanoemulsions (~1 µM) in phosphate-buffered saline (PBS) at pH 7.4 for size and 5 mM NaCl for charge.

### 2.4. Cell Viability of Resiquimod-GNE on M2-like RAW 264.7 Cells

M2-like RAW 264.7 cells were plated in a 96-well plate at a 10,000 cell per well density. After the cells adhered, they were treated with varied concentrations of resiquimod-GNE, GNE, and resiquimod. After 48 h, the cells were washed with Dulbecco’s phosphate-buffered saline (DPBS). Culture media containing 10% alamarBlue was added, and the plates were incubated for 3 h. The supernatant was then transferred to a 96-well black plate, and fluorescence was read at 560/590 nm (λex/λem) using a SpectraMax M2 plate reader (Molecular Devices, San Jose, CA, USA).

### 2.5. Confocal Imaging

RAW 264.7 cells were polarized to M2-like by treating them with 50 ng/mL interleukin-4 (IL-4) for 24 h. M2-like RAW 264.7 cells (100,000) were plated into 35 mm glass-bottom confocal dishes. The cells were then incubated with Nile Red-loaded GNE (1 μM) for 24 h, following which they were washed with DPBS, stained Hoechst 23342 for 15 min, and imaged with confocal laser scanning microscopy (CLSM) (40× objective). CLSM was performed using aA1R-TIRF Confocal Microscope (20× or 40× objectives) (Nikon Instruments Inc., Melville, NY, USA).

### 2.6. Pro-Inflammatory Marker Expression Assessment

M2-like RAW 264.7 cells (120,000 cells per well) were plated overnight in 6-well plates. The day after, 1 μM of resiquimod-GNE, GNE, and resiquimod alone were added. After 48 h, the cells were washed with DPBS, and cytokine expression levels were then assessed using quantitative reverse-transcription polymerase chain reaction (qRT-PCR) with a CFX Connect Real-Time PCR Detection System (Bio-Rad, Hercules, CA, USA) with iTag Universal SYBR Green Supermix. Briefly, 2 μg of RNA was extracted using the Pure Link RNA Mini kit (Ambion, Austin, TX, USA). Superscript IV Reverse Transcriptase (Invitrogen, Carlsbad, CA, USA) was then used to convert the RNA to cDNA, on which the qRT-PCR was performed to quantify the expression of tumor necrosis factor-alpha (TNF-α) and inducible nitric oxide synthase (iNOS), using the following primer sequences:

β-Actin (Forward): 5′-GATCAGCAAGCAGGAGTACGA-3′

TNF-α (Forward): 5′-CCTGTAGCCCACGTCGTAG-3′

iNOS (Forward): 5′-GTTCTCAGCCCAACAATACAAGA-3′

### 2.7. Flow Cytometry for Evaluation of Macrophage Repolarization

To further evaluate repolarization, cells were trypsinized and incubated with an allophycocyanin (APC)-labeled CD80 antibody at 60 ng per million cells in a flow buffer for 1 h and then quantified by flow cytometry. Flow cytometry was performed with LSR Fortessa Spectral Flow Cytometer (Becton Dickinson, Franklin Lakes, NJ, USA) and analyzed with FlowJo software.

### 2.8. Co-Culture Model

M2-like macrophages and GFP-U2OS cells were co-cultured in 35 mm confocal microscopy dishes at densities of 270,000 and 90,000 cells per dish, respectively. Then, 1 μM of resiquimod-GNE, GNE, and resiquimod only were added to the cells and incubated for 24 h. Following treatment, the cells were stained with Hoechst 33342 dye for nuclear staining for 15 min before undergoing CLSM (20× objective). The fluorescence intensity of GFP-U2OS cells was quantified using ImageJ.

## 3. Results and Discussion

Resiquimod-GNE was generated by loading resiquimod into the essential oil core of the gelatin nanoemulsion. The gelatin nanoemulsion platform utilizes gelatin as an amphiphilic biopolymer scaffold to encapsulate and stabilize hydrophobic payloads [41]. Gelatin was selected as a delivery scaffold due to its diverse hydrophilic and hydrophobic amino acid domains, which give it surfactant-like characteristics [42,43]. Eugenol, a major component of clove oil, was chosen as the interior oil phase of the nanoemulsion, given its biocompatibility [44,45,46] and ability to dissolve resiquimod.

**Figure 2:**
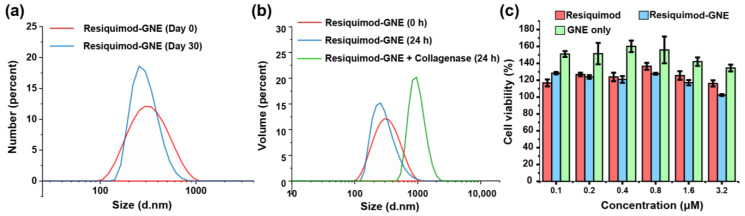
(**a**) Evaluation of the size of Resiquimod-GNE by dynamic light scattering (DLS) at room temperature for ≥30 d. (**b**) Stability studies of Resiquimod-GNE on incubation with collagenase type I enzyme at 37 °C. (**c**) Viability of M2-like RAW 264.7 cells after 48 h exposure to various concentrations of Resiquimod-GNE.

The Resiquimod-GNE produced were approximately 300 nm and remained stable in solution for a minimum of 30 d at room temperature (Figure 2a). However, we observed a notable increase in the size of the nanoemulsions in the presence of collagenase, indicating degradation of the gelatin scaffold followed by aggregation of the eugenol oil droplets (Figure 2b). The charge of the Resiquimod-GNE was 37.3 ± 1.9 mV, while that of GNE alone was 22.6.3 ± 0.8 mV.

Repolarization of macrophages without affecting their viability is important for immunotherapeutic reprogramming. Hence, we next examined the mammalian cell toxicity of Resiquimod-GNE against M2-like murine macrophages (M2-like RAW 264.7 cells). M2-like RAW 264.7 cells were treated with different concentrations of Resiquimod-GNE for 48 h, and the cell viability was assessed using an alamarBlue assay. Resiquimod-GNE demonstrated no cytotoxicity at concentrations relevant to therapeutic use (Figure 2c), with some hormesis observed for the GNE alone.

**Figure 3:**
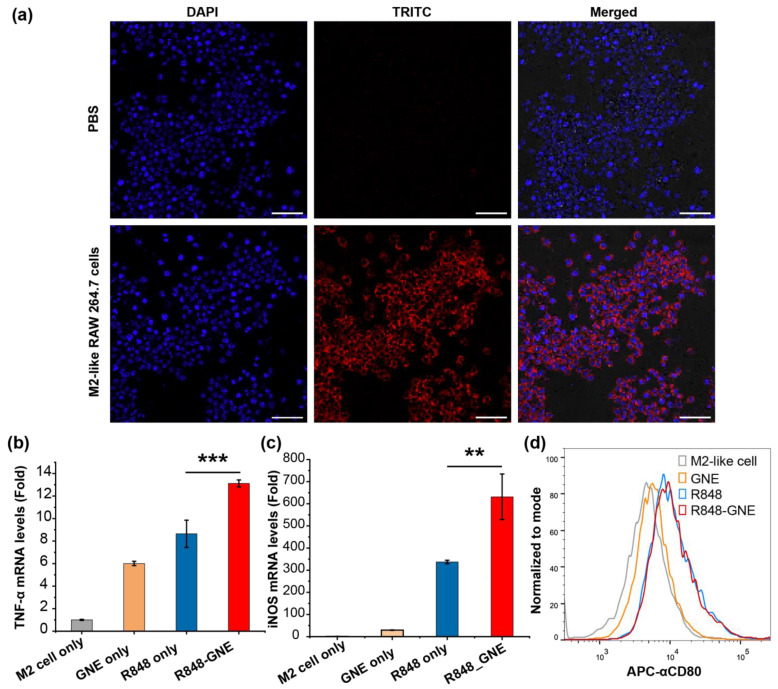
(**a**) CLSM images of M2-like RAW 264.7 cells incubated with the Nile Red-loaded GNE. Nuclei stained with Hoechst 33342 and visualized in a DAPI (blue) channel. Stained Nile Red was visualized in a TRITC (red) channel. The overlap of the blue and red channels is represented in pink. Scale bar = 50 μm. Quantitative analysis of (**b**) TNF-α and (**c**) iNOS expression levels using qRT-PCR. (**d**) Flow cytometry analysis of M1-related surface marker CD80 expression utilizing an APC-labeled CD80 antibody (APC-αCD80). Error bars represent the standard deviation (SD) from three experimental replicates. Data are presented as mean ± SD and analyzed using one-way ANOVA with Tukey’s multiple comparisons test. Statistical significance is indicated as *** (*p* < 0.001) and ** (0.001 ≤ *p* < 0.01), corresponding to a 99.9% confidence interval.

The ability of resiquimod to modulate macrophage repolarization strongly relies on its intracellular accumulation and interaction with the TLR 7/8 receptor. Hence, confocal laser scanning microscopy (CLSM) was employed to evaluate the ability of gelatin nanoemulsion to deliver hydrophobic payloads intracellularly and have them accumulate within M2-like macrophages. Nile Red, a hydrophobic red and fluorescent dye, was first loaded into the oil core, forming Nile Red-loaded GNE to track their intracellular transport. Nanoemulsions were incubated for 24 h with M2-like RAW 264.7 cells stained with Hoechst 33342 nuclear dye. As shown in Figure 3a, Nile Red-loaded GNEs effectively delivered their hydrophobic payload, Nile Red, into macrophages, as demonstrated by the red fluorescence distributed throughout the cytosol of the cells.

Motivated by the findings from imaging studies, we proceeded to examine the repolarization of M2 to M1-like macrophages via the intracellular delivery of the resiquimod-loaded nanoemulsion to Resiquimod-GNE through analysis of the mRNA expression of tumor necrosis factor-alpha (TNF-α) and inducible nitric oxide synthase (iNOS), two pro-inflammatory cytokines. The quantitative reverse transcription-polymerase chain reaction (qRT-PCR) results showed that cells treated with Resiquimod-GNE expressed significantly higher levels of TNF-α and iNOS compared to cells treated with resiquimod or GNE alone (Figure 3b,c), indicating that loading resiquimod into the nanoemulsion promoted M2 to M1-like repolarization.

We analyzed CD80 expression, a surface marker associated with M1-like macrophages, to further establish repolarization to M1-like phenotypes. Being a co-stimulatory signaling molecule essential for T-cell activation, CD80 plays an important role in the initiation of cell-based immunotherapy [47]. Flow cytometry revealed that M2-like RAW 264.7 cells incubated with Resiquimod-GNE exhibited a two-fold increase in CD80 receptor expression compared to the negative control groups (Figure 3d). Collectively, these results indicated that Resiquimod-GNE rapidly penetrated and accumulated inside M2-like macrophages, leading to efficient repolarization into M1-like phenotypes.

**Figure 4:**
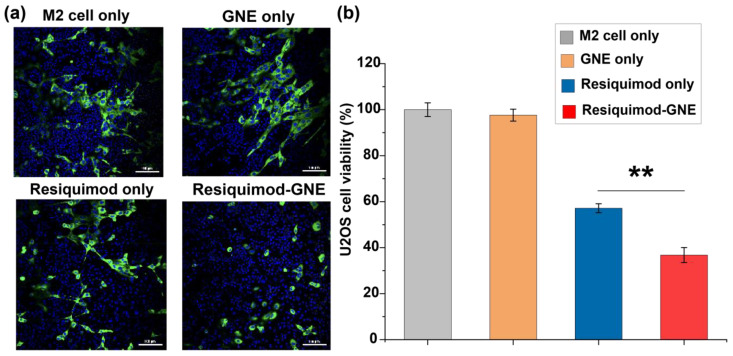
(**a**) CLSM images of GFP-U2OS and M2-like RAW 264.7 cells in a co-culture model for assessment of the anticancer potential of re-educated macrophages mediated by Resiquimod-GNE. Nuclei stained with Hoechst 33342 (visible in blue). Scale bar = 100 μm. (**b**) Cancer cell viability is assessed by measuring the intensity of green fluorescence. Data are presented as mean ± SD from three experimental replicates and analyzed using one-way ANOVA with Tukey’s multiple comparisons tests. Statistical significance is indicated as ** (0.001 ≤ *p* < 0.01) , corresponding to a 99.9% confidence interval (CI).

The anticancer potential of macrophages re-educated by GNEs was assessed using a co-culture model of M2-like RAW 264.7 and osteosarcoma U2OS cancer cells expressing green fluorescent protein (GFP-U2OS cells). The co-cultured cells were treated with Resiquimod-GNE and appropriate controls for 24 h before performing CLSM. GFP fluorescence was used as a measure of cancer cell viability. Substantially reduced green fluorescence intensity was detected in cells with Resiquimod-GNE and resiquimod alone treatment groups, consistent with macrophage-induced cell killing. Notably, significantly greater efficacy was observed with Resiquimod-GNE relative to resiquimod alone, demonstrating the efficiency of the GNE platform. As expected, GNE alone had no notable impact on the cancer cells, with fluorescence levels remaining comparable to those of the control. (Figure 4).

## 4. Conclusions

In summary, we report an all-natural nanoemulsion scaffold that effectively delivers a hydrophobic TLR 7/8 agonist into macrophages. The nanoemulsion was generated using all-natural components, with resiquimod as the agonist. The resulting nanoemulsions showed excellent solution stability as well as biodegradability. The Resiquimod-GNE were effectively uptaken into the M2-like cells, allowing efficient delivery of resiquimod and promoting the repolarization of TAMs to an anticancer phenotype through activation of TLR 7/8 agonists. By reprogramming TAMs to exhibit a tumoricidal M1 phenotype, this nanoemulsion platform could significantly enhance the efficacy of existing cancer treatments and contribute to overcoming resistance to chemotherapy. Moreover, combining the repolarization of TAMs with chemotherapy can offer an integrated immuno-chemotherapy strategy, potentially improving therapeutic efficacy.

## Data Availability

The raw data supporting the conclusions of this article will be made available by the authors upon request.
